# Neuronal and Glial Communication via Non-Coding RNAs: Messages in Extracellular Vesicles

**DOI:** 10.3390/ijms24010470

**Published:** 2022-12-28

**Authors:** Davide Marangon, Juliana Helena Castro e Silva, Davide Lecca

**Affiliations:** Laboratory of Molecular and Cellular Pharmacology of Purinergic Transmission, Department of Pharmaceutical Sciences, Università degli Studi di Milano, 20133 Milan, Italy

**Keywords:** extracellular vesicles, cell-to-cell communication, microRNA, long non-coding RNA, circRNA, neurons, microglia, astrocytes, oligodendrocytes

## Abstract

Extracellular vesicles (EVs) have been increasingly recognized as essential players in cell communication in many organs and systems, including the central nervous system (CNS). A proper interaction between neural cells is fundamental in the regulation of neurophysiological processes and its alteration could induce several pathological phenomena, such as neurodegeneration, neuroinflammation, and demyelination. EVs contain and transfer complex molecular cargoes typical of their cells of origin, such as proteins, lipids, carbohydrates, and metabolites to recipient cells. EVs are also enriched in non-coding RNAs (e.g., microRNAs, lncRNAs, and circRNA), which were formerly considered as cell-intrinsic regulators of CNS functions and pathologies, thus representing a new layer of regulation in the cell-to-cell communication. In this review, we summarize the most recent and advanced studies on the role of EV-derived ncRNAs in the CNS. First, we report the potential of neural stem cell-derived ncRNAs as new therapeutic tools for neurorepair. Then, we discuss the role of neuronal ncRNAs in regulating glia activation, and how alteration in glial ncRNAs influences neuronal survival and synaptic functions. We conclude that EV-derived ncRNAs can act as intercellular signals in the CNS to either propagate neuroinflammatory waves or promote reparative functions.

## 1. Introduction

The complex morphology and function of the central nervous system (CNS) demands tightly orchestrated mechanisms of regulation and control of gene expression throughout the entire lifespan of an organism. This tight regulation takes place in neural stem cells (NSCs), neurons and glial cells at various developmental stages and allows to control neurogenesis, synaptogenesis, neurite outgrowth, neuronal maturation, synaptic plasticity, and myelination [1,2,3,4]. Non-coding RNAs such as microRNAs, long non-coding RNAs (lncRNAs), and circular RNAs (circRNAs) have fundamental roles in regulating these processes at post-transcriptional level [5,6,7].

MiRNAs are small RNA molecules that bind to the 3′UTR of their target transcripts, resulting in mRNA degradation or translational repression [5]. Despite them not being the first-discovered ncRNA class, the number of studies on miRNAs indexed on Pubmed is larger than all the studies on circRNAs and lncRNAs together. Indeed, in biomedical and clinical research, miRNAs are considered important factors for developing new diagnostic and therapeutic strategies. LncRNAs, initially considered as “junk RNA”, are now known to contribute to regulation of various physiological and pathological processes, and their number (more than 30,000) exceeds that of known mRNAs [6]. Thanks to their complex and thermodynamically stable secondary structure, lncRNAs can interact with DNA, RNA, and proteins, modulating chromatin structure and transcription, affecting RNA splicing, stability, and translation [8]. CircRNAs are a class of covalently closed circular ncRNAs, formed when a splice donor and upstream acceptor from a linear RNA are linked together, in a process known as backsplicing [7]. Recent evidence shows that circRNAs play sophisticated biological roles, including regulation of pre-mRNA splicing, RNA binding protein sequestration, and IRES-mediated CAP-independent translation to produce short peptides [9]. Interestingly, both circRNAs and lncRNAs can bind to miRNAs to sequester, or “sponge” them [10], leading to the generation of a complex network constituted by multiple circRNA/lncRNA-miRNA-mRNA axes, which could ultimately regulate hundreds of genes.

Originally, ncRNAs were described as cell-intrinsic regulators that maintain cell homeostasis, and their alteration was specifically associated with the onset of several pathological conditions, including neurodegenerative diseases. However, in the last decade, they have been demonstrated to play crucial roles in cell-to-cell communication in a paracrine fashion, similarly to soluble factors. Both neurons and glial cells release ncRNAs stored into extracellular vesicles (EVs), whose structure and functions are increasingly gaining attention in the scientific community as both extracellular messengers and peripheral biomarkers.

EVs are lipid bound vesicles released by most cell types and they are characterized by a specific set of proteins, lipids, and nucleic acids, most of which are ncRNAs [11]. Based on their biogenesis, they can be broadly categorized into microvesicles (100–1000 nm), and exosomes (30–120 nm). While the former derive from membrane budding, the latter are secreted from the endosomal compartment. Even though the amount of ncRNAs into EVs could reflect their cellular abundance [12], deep sequencing revealed that, generally, EVs have a distinct profile compared to their parent cells, suggesting a regulated sorting rather than random packaging [13,14]. Other studies also suggest that the abundance of ncRNAs in EVs does not directly correlate with their intracellular abundance and that their biological relevance may be cell type dependent [15]. For example, it has been proposed that exosomes from mesenchymal stem cells mainly act through their protein rather than RNA cargoes [16]. However, it is worth mentioning that several stimuli and pathological contexts have been associated with both increased production of exosomes [17,18,19,20] and alteration of their ncRNA content [21,22], thus reinforcing the potential relevance of ncRNA-based mechanisms in cell-to-cell communication. 

EVs released by NSC, neurons, and glia have been shown to be selectively taken up by the neighboring cells, where their cargo can induce proliferation, differentiation, migration, and modulate inflammatory response during acute or chronic injury, depending on their very specific cargo [11]. Thus, it is not surprising that deregulated expression of specific ncRNAs in EVs is an important factor in various CNS pathologies, including neurodevelopmental, neurodegenerative, and neuroimmunological disorders, brain tumors, and psychiatric diseases.

In this review, we describe some of the mechanisms by which NSC, neuronal and glial EVs, and their ncRNA cargoes communicate in physiological and pathological conditions. We also envisage the potential translatability of these findings into disease diagnosis, monitoring, and treatment.

## 2. Effect of NSC-Derived ncRNAs on Neurogenesis

In the last decade, NSC therapy has shown promising results for brain repair after injury or disease [23], but safety issues have hindered their clinical application. Although transplanted NSCs have the potential to engraft, differentiate, and regenerate damaged brain tissue, their functional properties have been attributed mostly to the release of trophic factors in the damaged CNS, suggesting that NSC-EVs, and their RNA cargo, may show similar regenerative effects. From this perspective, a recent paper investigated their composition through small RNA sequencing and proteomics, to define their biological activity and neuroreparative properties [24]. Among the miRNAs identified in NSC-EVs, several (e.g., miR-320a, miR-103a-3p, miR-21-5p, miR-26a-5p, miR-320b, miR-30a-3p, miR-181a-5p, miR-191-5p) were already known to contribute to various aspects of brain functions and repairing after injury. Pathway analysis for the most abundant miRNAs showed that NSC-EVs carry a cargo of miRNAs that are important for multiple signaling pathways, such as fatty acid biosynthesis and metabolism, extracellular matrix-receptor interaction, adherens junction, cell cycle, p53, transforming growth factor-β (TGF-β), and thyroid hormone signaling. Intranasal administration of NSC-EVs resulted in their rapid incorporation by virtually all neurons and microglia in all the CNS regions and enhanced hippocampal neurogenesis in adult rats [24]. Nevertheless, this study did not assess the contribution of specific miRNAs and lncRNAs on these effects, thus gain-of-function and loss-of-function studies on specific ncRNAs in NSC-EVs are needed to define their role. In a similar context, a recent paper investigated the role of a specific circular RNA, namely Acbd6, upregulated in exosomes produced by denervated hippocampi following fimbria–fornix transection and predominantly localized in the cytoplasm of NSCs [25]. Exosomes derived from denervated hippocampus, as well as over-expression of circAcbd6, facilitated the differentiation of NSCs into neurons and cholinergic neurons, and significantly improved the learning and memory ability in a rat model of cholinergic injury. Further molecular studies showed that circAcbd6 acts as an endogenous miR-320-5p sponge to inhibit its activity, resulting in increased expression of the oxysterol-binding protein–related protein 2 (Osbpl2) in NSCs and the subsequent enhancement of their differentiation [25]. The neurogenic potential of NSC-derived EVs is also mediated by miR-9-5p, which was identified as the most abundantly expressed miRNA. According to this, the pro-neurogenic and pro-differentiative effects were abrogated by depleting exosomal miR-9-5p. Further molecular studies showed that these effects are mediated by its interaction with several genes involved in repressing neurogenesis, such as Hes1 and REST [26]. Despite these studies highlighting the potential of EVs as an alternative therapeutic strategy to NSC transplantation, further studies are expected to demonstrate their superiority in terms of efficacy, safety, and practicability.

## 3. Role of Neuronal EV-Derived ncRNAs in Neuron-to-Glia Communication

The release of EVs has been demonstrated both in developing and terminally differentiated neurons. Neuron-derived EVs (NDEVs) contain some neuron-specific markers, including L1 cell adhesion molecule (L1CAM) and the GluR2/3 subunits of glutamate receptors [11]. Despite it having been shown that, upon synaptic activation, EVs released by cortical neurons are selectively absorbed by neurons [27], several recent studies suggested that NDEVs can also be taken up by glial cells, influencing their functions through ncRNA-based mechanisms. A summary of the studies described in the following paragraphs is reported in Table 1.

### 3.1. Effect of Neuron-Derived ncRNAs on Microglia Functions

A recent study reported the importance of neuron–microglia communication in the maintenance of the deactivated phenotype of microglia in the normal CNS [28]. Despite the mechanism involved in such deactivation is still largely unknown, this study highlighted the role of miR-124-3p, a well-known neuron-enriched miRNA, which is downregulated in activated microglia and is sufficient to deactivates bone marrow-derived macrophages in vitro [29]. Ex vivo isolated adult microglia showed high levels of miR-124-3p, which then decreased after several weeks in vitro. These results may imply that adult microglia express miR-124-3p only in the CNS microenvironment [28] and suggest that miR-124 expression depends on the paracrine interaction with, or the transfer from, other CNS cells. Accordingly, microglia treated with neuron-conditioned medium started expressing miR-124-3p.

**Table 1:** Summary of neuronal EV-derived ncRNAs, their targets, and effects.

Source	Recipient Cells	Neuronal ncRNAs	ncRNA Targets	Mechanism of Action	Reference
Mouse cortical neurons	Mouse microglia	miR-124-3p	CEBPα-PU.1 and NFκB	Microglia deactivation in the normal CNS	[28]
Mouse neurons	Mouse microglia	miR-124-3p	MYH9 and NFκB	Suppressing microglia activation in SCI	[30]
Rat PC12 cells	Mouse microglial BV2 cell line	miR-9-5p	SOCS2	Promotion of the M1 phenotype in MDD	[31]
Mouse cortical neurons	Mouse cortical astrocytes	miR-124-3p	GLT1-binding miRs, CREB	Regulation of synaptic functions in CNS	[32]
Mouse NSC-34 neuronal cell line	Mouse cortical astrocytes	miR-218-5p	GLT1	Alteration of glutamate uptake and astrogliosis	[33]
Rat cortical neurons	Rat cortical astrocytes	miR-181c-3p	CXCL1	Reduction of neuroinflammation	[34]
Mouse CATH.a neuronal cell line	Mouse C8-D1A astrocytic cell line	lncRNA H19	miR-18a/VEGF axis	Alteration of BBB permeability	[35]

Along with miR-124-3p, miR-9-5p is a key regulator of neuronal differentiation [36] that regulates macrophage activation outside the CNS [37], thus likely playing immunomodulatory functions in the CNS microenvironment as well. It has been shown that neuronal miR-124-3p and miR-9-5p accumulate on the surface of neurons binding to glycolipids and glycoproteins, and they can be released by electrically active neurons. Interestingly, further analysis demonstrated that extracellularly released miR-124-3p and miR-9-5p formed complexes with HDLs, whereas miR-124-5p and miR-9-3p, the passenger strands, were enriched in the exosomal fraction [28].

Neuronal ncRNAs were not only shown to be relevant for the maintenance of microglia function in the healthy CNS, but also for the regulation of microglial immune responses after injury. Administration of either EVs isolated from untreated and miR-124-3p over-expressing neurons were able to promote functional behavioral recovery in a contusive spinal cord injury in vivo model by suppressing the activation of neurotoxic microglia [30]. In agreement with this, microglia treated with miR-124-3p-enriched NDEVs showed a reduction in pro-inflammatory cytokines release and attenuated iNOS immunofluorescence. The mechanism of action involves the direct interaction of miR-124-3p with myosin heavy chain 9 (MYH9), and ultimately the activation of the PI3K/Akt and inhibition of the NF-κB pathways [30]. Despite miR-124-3p and miR-9-5p play similar role in neuronal cells, another recent study showed that they may exert an opposite activity on microglia activation in some pathological context [31]. In this study, a neuron–microglia transwell co-culture system was used to demonstrate the exosomal transfer of miR-9-5p from neurons to microglia, which led to up-regulation of miR-9-5p and an increase in the number of M1 microglia. Concomitantly, the intracellular levels of the inflammatory factors IL-1β, IL-6 and TNF-α were found significantly upregulated. These effects were completely abolished pretreating neuronal cells with GW4869, an exosome biogenesis inhibitor. Interestingly, miR-9-5p was also found upregulated in exosomes isolated from the serum of major depressive disorder (MDD) patients compared to healthy subjects [31]. Over-expression of miR-9-5p in MDD mice enhanced M1 polarization and increased depression-like behavior by suppressing SOCS2 expression and activating the JAK/STAT3 pathways; conversely, miR-9-5p inhibition alleviated depression in MDD mice. These results suggest that exosomal miR-9-5p could serve as a potential messenger in the pathogenesis of MDD and may represent a marker for MDD diagnosis.

### 3.2. Effect of Neuron-Derived ncRNAs on Astrocyte Functions

Despite the recognized role of astrocytes in regulating synapse function, molecular pathways that regulate the neuron-astroglia functional unit are largely undefined. In this context, several recent studies highlighted that NDEVs, and especially their RNA cargo, may exert an essential role in regulating astrocyte functionality and activation. As previously reported for microglial cells, despite the expression of miR-124-3p is negligible in pure astrocyte cultures, its expression was 12-fold higher in astrocytes isolated by FACS from neuron-astrocyte primary cocultures [32]. Interestingly, this upregulation was not coupled to an increase of the relative primary transcript (pri-miR-124), which was found as low as that in astrocytes alone, suggesting that miR-124-3p upregulation in astrocytes is a result of miR-124-3p transfer from neurons. This transfer was also demonstrated in vivo taking advantage of the retrograde transport of sciatic nerves to spinal motor neurons (SMNs) and of transgenic mice that allow to selectively deliver peripheral injected Cy5-miR-124-3p into SMNs and to consequently label SMN-derived exosomes [32]. In this model, Cy5-miR-124-3p labeling was found inside SMNs and neighboring astrocytes, either co-localizing with CD63, an indication of exosome association, or by itself. The in vivo transfer was validated by daily administration of GW4869, which significantly decreased miR-124-3p levels in isolated astrocytes by 80% compared to sham controls. Exosomal miR-124-3p upregulated the predominant glutamate transporter GLT1 in astrocytes by suppressing the expression of several GLT1-inhibiting miRNAs, suggesting that neuronal activity-dependent release of exosomes and their RNA cargo, from post-synaptic soma or dendrites is implicated in regulating synaptic functions. A similar mechanism of action was recently demonstrated for another neuron-enriched miRNA, namely miR-218-5p, in the context of amyotrophic lateral sclerosis (ALS). miR-218-5p was found in the CSF, in protein-bound form, after motor neuron loss in a rat model of ALS [33]. Further studies showed that the uptake of miR-218-5p in astrocytes abolished the expression of the GLT1, whose loss has already been well-described in both ALS patients and animal models [38]. This study also showed that the effect of miR-218-5p on astrocytes could be blocked in vitro and in vivo by administering a specific anti-miR [38].

miR-181c is another neuron-enriched miRNA that plays a role as a modulator of the gene network linked to cortical neuronal maturation and that was shown to act as an important messenger in pathological conditions. Over-expression of miR-181c has also been reported to protect neurons against apoptosis mediated by activated microglia via TNF-α suppression [39]. Alteration of exosomal miR-181c-3p levels were associated to a worse inflammatory response in ischemic brain injury (IBI) [34]. This study suggested that neuronal miR-181c could be necessary to fine-tune the expression levels of the astrocytic chemokine CXCL1, a member of the C-X-C family with the ability to attract neutrophils into the injured CNS [40], and control CNS inflammation [34]. The expression of miR-181c-3p was found remarkably lower, while the expression of CXCL1 was significantly higher in an IBI animal model. The relationship between neuronal miR-181c-3p and astrocytic CXCL1 was investigated by an in vitro IBI model where exosome isolated from OGD-treated cortical neurons were put in contact with astrocytes. Following OGD, a lower expression of miR-181c-3p was found in both cortical neurons and exosomes, suggesting that IBI might lead to an inhibited release of exosomal miR-181c-3p from cortical neurons and to an increased neuroinflammation. Accordingly, treatment of astrocytes with either miR-181c-3p containing exosomes isolated from healthy neurons, or miR181c mimics, reduced the expression of CXCL1 and other pro-inflammatory markers [34]. 

One of the pathophysiological features of ischemic stroke is disruption of the blood–brain barrier (BBB), mainly constituted of endothelium and astrocytes, that significantly contributes to brain injury and subsequent neurological impairment [41]. In this context, a very recent study explored the pathophysiological function of a neuron-derived lncRNA, namely H19, in regulating the crosstalk between astrocytes and endothelial cells, and BBB integrity after cerebral ischemic stroke [35]. In this study, OGD/R increased H19 levels in neuronal exosomes, which added to the culture medium of the astrocyte and endothelial cell co-culture increased endothelial permeability. Of note, the expression of H19 inversely correlated with that of miR-18a, in line with previous demonstrations that miRNAs belonging to the miR-17-92 cluster are inhibited by this lncRNA [42], and directly correlated with VEGF, a potential direct target of miR-18a [43] associated with angiogenesis and BBB breakdown, suggesting the existence of a H19/miR-18a/VEGF axis that regulates BBB permeability after stroke. In line with this, OGD coupled to inhibition of H19 in neurons reduced VEGF levels in astrocytes, due to reduced sponge activity of the lncRNA on astrocytic miR-18a, and ultimately reduced permeability and tight junction protein expression in endothelial cells. In contrast, the simultaneous inhibition of H19 in neurons and miR-18a in astrocytes increased these parameters and the addition of a VEGFR2-blocking antibody completely reversed this effect [35]. 

### 3.3. Effect of Neuron-Derived ncRNAs on Oligodendrocytes

To date, the role of EV-derived ncRNAs in the communication between neurons and oligodendrocytes is largely unexplored. However, the evidence that NDEVs can be acquired by other glial cells and that several ncRNAs have shown to deeply influence oligodendrocyte behavior strongly suggest that neuron-derived ncRNAs could play a significant role in regulating oligodendrocyte development, especially in pathological conditions. For example, recent studies from our group showed that miR-125a-3p is expressed either by neurons and oligodendrocytes and that its over-expression leads to myelination impairment [44,45,46]. These results coupled to the notion that NDEVs were also found enriched in miRNAs from miR-125 family [47], may imply that in neurodegenerative conditions a miR-125a-3p-mediated interplay between neurons and oligodendrocytes contribute to limit lesion repair upon injury. Other neuron-enriched miRNAs, such as miR-9 and miR-124, were found abundantly present in NDEVs, upregulated in neurodegenerative conditions [48,49] and downregulated during oligodendrocyte differentiation. miR-9-5p expression was found inversely correlated with that of peripheral myelin protein PMP22, a protein expressed in Schwann cells (which lack miR-9) but absent in oligodendrocytes [50], whereas miR-9-3p was shown to negatively regulate oligodendrocyte lineage gene 1 (Olig1) in brain tissues during hypoxic-ischemic brain damage [51]. Increased miR-124-3p levels were instead associated with demyelination of the hippocampus in human MS samples, cuprizone-induced demyelination [52] and demyelination of the prefrontal cortex (PFC) in a mouse model of autism spectrum disorder (ASD). In this last model, miR-124-3p was found aberrantly expressed in oligodendroglial cells, suggesting a possible EV-mediated neuron-to-oligodendrocyte transfer of miR-124-3p responsible for myelination impairment [53]. Accordingly, expression of a miR-124-3p sponge in the PFC of ASD mice, either ubiquitously or specifically in oligodendrocytes, rescued the myelin defects, and improved their performance on social behaviors. Nevertheless, in the ventral zebrafish hindbrain, loss of miR-124-3p decreased oligodendrocyte cell numbers and myelination of axonal projections [54], suggesting that ncRNA-mediated axonal–glial interactions may play a different role during neural development.

## 4. Role of Glial EV-Derived ncRNAs in Cell-to-Cell Communication

### 4.1. Effect of Microglial EV-Derived ncRNAs on Neuroinflammation

In the CNS, microglia are essential players, highly reactive to extracellular stimuli and continuously communicating with the neighboring cells [55,56]. Based on the extracellular microenvironment, microglia can acquire either a pro-inflammatory M1 phenotype that triggers phagocytosis of pathogens and cell debris, or an anti-inflammatory M2 phenotype that promotes tissue repair [57]. After activation, they orchestrate CNS inflammatory response by secreting both soluble factors such as cytokines and chemokines [8], and EVs, which also contain signaling molecules and ncRNAs [58]. Several recent studies have shown that microglia activation state directly influences the ncRNA composition of EVs [59,60,61], which in turn can regulate the expression of neuronal synaptic proteins, enhance excitatory neurotransmission, propagate an activation signal to astrocytes and to other microglia, and control OPC recruitment and differentiation.

In vitro, the exposure of microglia to different pro-inflammatory stimuli leads to enrichment of miRNAs in EVs that contribute to the spread of inflammation in the brain parenchyma. Interestingly, when stimulated with either β-amyloid or a pro-inflammatory cocktail (IL-1β, TNF-α, and IFN-γ) the miRNA composition was found comparable, suggesting that degenerative and inflammatory contexts may share common alteration in miRNA-mediated mechanisms [59]. EVs derived from pro-inflammatory microglia (M1-EVs) were found particularly enriched in miR-146a-5p, that directly targets neuroligin1 (Nlg1) and synaptotagmin 1 (Syt1), miR-181a, and miR-223-3p, targeting the glutamate metabotropic receptors GluR2, and GluN2B [59]. The same miRNAs were also found in the CSF of multiple sclerosis patients, providing a possible link between microglia activation, enhanced EV production, and cognitive symptoms in MS patients. Of note, miR-146a-5p is the only one not expressed in neurons and, in addition, the first ncRNA shown to be taken up by neurons in a glia-to-neuron communication [59]. The neuronal intake of microglia-derived miR-146a-5p specifically reduced frequency and amplitude of excitatory postsynaptic currents and the dendritic spine density in vitro. These effects were not observed in neurons with a mutant Ngl1 form lacking the 3′-UTR, thus validating the role of microglia-derived miR-146a-5p in the process [59]. A recent study showed that microglial miR-146a-5p is also enriched in exosomes isolated from the serum and hippocampus of rats subjected to chronic unpredictable mild stress (CUMS), a model of depression, affecting neurogenesis [62]. In neuropsychiatric disorders, such as major depression, it is suggested that the neurogenic niches are reduced in size due to a neurogenesis impairment [63]; however, the cellular mechanisms that lead to this impairment are largely unknown, especially regarding the contribution of glial ncRNAs. When overexpressed in vivo, miR-146a-5p induced a depressive-like phenotype in rats not exposed to chronic stress, whereas the miR-146a-5p knock-down in exposed rats induced an amelioration of the symptoms. In vitro, the treatment of neurons with M1-EVs induced proliferation and migration impairments, which were reverted by miR-146a-5p knock-down, indicating that exosomes derived from microglia can deliver miR-146a-5p to neurons to regulate neurogenesis [62]. The authors have demonstrated that the mechanism consists in the direct repression of two neurogenic factors: Krüppel-like factor (KLF4) and cyclin-dependent pathway (CDKL5). These results suggest that microglia-derived miR-146a-5p can function as a crucial signaling mediator during neurogenesis in depression and represent a novel therapeutic target in this disease [62]. Furthermore, the authors also identified a circRNA, namely circANKS1B, downregulated within dentate gyrus regions of CUMS rat and presenting seed sequence matching for miR-146a-5p. The over-expression of circANKS1B increased the number of newly developed neurons and the levels of KFL4 and CDKL5, indicating that circANKS1B can function as a sponge for miR-146a-5p to repress its activity, thus representing a new potential therapeutic target to foster neurogenesis in depression [62].

On the other hand, a pro-regenerative environment can result in the enrichment in EVs of miRNAs that induce reparative functions. Treatment of mice subjected to transient middle-cerebral artery occlusion (tMCAO) with EVs isolated from microglial cells previously stimulated with IL-4 (M2-EVs) reduced brain atrophy, promoted sensorimotor and cognitive functional recovery, enhanced white matter structural remodeling and functional repair, when compared to mice treated with vesicles from non-stimulated microglia [60]. The same study also showed that these vesicles were incorporated by OPCs and promoted their proliferation both in vivo and in vitro and that these effects depend on the downregulation of Olig3, induced by miR-23a-5p, the most enriched miRNA in M2-EVs. Interestingly, miR-23a-5p knock-down in M2-EVs abolished the vesicles’ positive effects on tMCAO, suggesting that the miR-23a-5p-based glia-to-glia communication is essential in reducing ischemic injury and improving neurological functional recovery [60].

In the same context, M2-EVs were also shown to reduce the size of the glial scar astrogliosis, neuronal apoptosis, and infarct volume. Recent studies suggested that these effects are likely mediated by their enrichment in miR-124 and miR-137, two neuron-specific miRNAs that in physiological conditions are not expressed by glial cells [64,65]. Indeed, miR-124 knock-down in M2-EVs completely abolished their beneficial effects on astrocytes, suggesting that M2-EVs reduce mouse brain atrophy volume and improve neurobehavioral outcomes by transferring miR-124. The mechanism involves its direct interaction with STAT3, which is shown to be increased in tMCAO [61]. Of note, treatment with M2-EVs decreased p-STAT3 and increased Notch1 expression—a transcription factor shown to be activated in hypoxia and stroke and to promote neuronal survival and to determine the fate of neurons [66]—together with Sox2, a key player in the astrocytic reprogramming to neural progenitors [61], suggesting that miR-124-3p might also be involved in astrocyte reprogramming to neural progenitor cells after ischemic stroke. On the other hand, the protective effect of M2-EVs on neurons after tMCAO was partially reduced when miR-137 was knocked down in M2 microglia [65]. Interestingly, also in this case, the mechanism involves the direct modulation of Notch1.

M2-EVs and miR-124 reduced neuroinflammation and promoted neuronal viability also in a traumatic brain injury (TBI) model. In this context, miR-124-3p simultaneously targets Rela [67], an inhibitory transcription factor of ApoE that promotes β-amyloid proteolytic breakdown, FIP200 [68], a protein that plays a key role autophagosome formation and in trauma-induced autophagy, and PDE4B [64], a protein involved in neuroinflammation and mTOR signaling activation.

**Figure 1:**
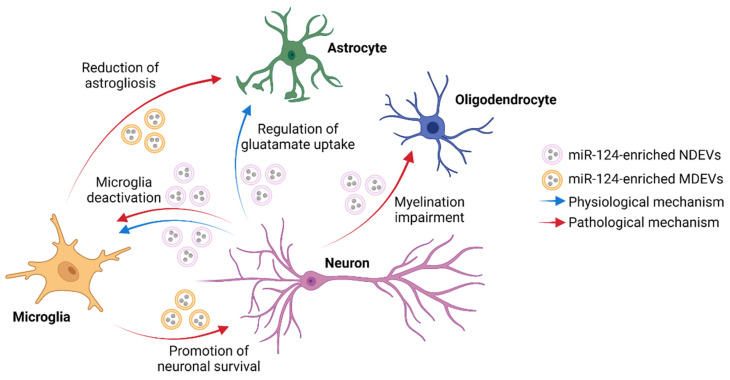
Schematic representation of the potential neuron-glia interactions mediated by vesicular exchange of miR-124 in both physiological and pathological conditions. NDEVs = neuron-derived EVs; MDEV = microglia-derived EVs. Created with BioRender.com.

Altogether, these studies suggest that miR-124-mediated communication could represent a pro-regenerative mechanism that aims at restoring miR-124 levels, reducing neuroinflammation and promoting neuronal survival and repair in the CNS (Figure 1). Treatment with microglial EVs enriched with miR-124 and miR-137 represents a promising therapeutic strategy for the treatment of brain ischemia and TBI. A summary of the studies described in this paragraph is reported in Table 2.

### 4.2. Effect of Astrocytic EV-Derived ncRNAs on Neuroinflammation

Astrocytes play an essential role in the homeostasis of CNS. Among their many functions, they are responsible for CNS detoxification, regulation of neurotransmitter levels, and for providing energetic support to other neural cells [69]. Astrocyte-derived EVs (ADEVs) and their ncRNA cargo were shown to directly modulate target transcripts in microglia, oligodendrocytes, and neurons in health and disease [70,71].

As described for microglia, exposure to inflammatory cytokines can affect the content of ADEVs, thus altering neuron communication [70,72]. ADEVs stimulated with TNF-α (TNFα-ADEVs) and with IL-1β (IL-1β-ADEVs) were shown to reduce the total neuronal area, neurite length, dendritic number and complexity, and the formation of nodes from primary hippocampal neurons [70]. Importantly, these effects were found even after antagonizing the cytokine receptors in neurons, which excludes the possible direct effect of cytokines. TNF-α- and IL-1β-ADEVs showed commonly enriched miRNAs, suggesting that the inflammatory stimuli generate convergent ncRNA changes. In particular, miR-125a-5p and miR-16-5p were more than two-fold enriched. The authors validated the transmembrane tyrosine kinase receptor for neurotrophin 3 (NT-3) and its downstream effector Bcl-2 as their direct targets, showing that miRNAs from pro-inflammatory ADEVs modulate the neurotrophin signaling pathway in neurons [70]. In line with this study, exposure of astrocytes to lipopolysaccharide (LPS) induced the generation of EVs (LPS-ADEVs) that induced a detrimental effect on neurons [73]. Also in this case, the ADEVs showed significant miRNA alterations, reinforcing the existence of a miRNA-based communication from astrocytes toward neurons in neuroinflammatory conditions. Interestingly, LPS-ADEVs were found enriched in miR-34a that hampers neuronal functions by targeting Bcl-2 [73]. The authors also showed that treatment with LPS-ADEVs in a rat model of Parkinson’s disease worsened the loss of dopaminergic neurons in the substantia nigra, which was partially reverted after blocking the production of miR-34a in astrocytes. However, at a later timepoints, the blockade of miR-34a production could not counteract the loss of dopaminergic neurons [70], suggesting that other miRNAs found in the ADEVs and not analyzed in the paper may play an essential role in the damage [73]. Together, these data indicate that inflammation induces a reorganization in the EV cargo and secretion, which in turn, impairs neuronal functions.

NcRNAs derived from astrocytic EVs can also play an important protective role on neurons and positively modulate neuronal synaptic activity [72,74,75]. In this context, some miRNAs were exclusively found in astrocytes and were not secreted through EVs, whereas some miRNAs, such as miR-190b, were highly abundant in ADEVs and were shown to exert their function in neighboring cells [76]. miR-190b was shown to partially improve the viability of the neuronal cell line HT-22 after OGD, suggesting a protective role of this ncRNA. Instead, in the same injury model, its knock-down in ADEVs induced a more relevant increase in the expression of pro-inflammatory markers in neurons, such as TNF-α, IL-1β and IL-6, suggesting that miR-190b protects neuronal function by modulating inflammation. miR-190b was predicted to target the Autophagy Related 7 protein (Atg7), whose activity is essential for the autophagosome membrane formation [75]. Accordingly, it was shown that Atg7 overexpression was detrimental for neuronal cell viability and had a role in inducing cell apoptosis, whereas Atg7 inhibition mediated by miR-190b enhanced neuronal viability after OGD via autophagy suppression [75].

NcRNAs derived from ADEVs can also positively interfere on NF-κB and autophagy pathways in damage conditions. NKILA, the nuclear transcription factor NF-κB interacting lncRNA, was found downregulated in isolated neuronal cultures after mechanical injury compared to non-damaged neurons. However, when co-cultured with astrocytes NKILA levels were re-established [77], suggesting that its uptake from astrocytes could help restoring neuronal viability [77]. The authors demonstrated that NKILA binds to miR-195 (repressing its activity), which was previously reported to inhibit autophagy and to target the Nod-like receptor NLRX1. Accordingly, miR-195 overexpression reduced, whereas NKILA overexpression increased the activity of NLRX1. The levels of NLRX1 were also increased when neurons were treated with NKILA-rich ADEVs compared to astrocyte EV-free media, thus confirming the involvement of the NKILA/miR-195/NLRX1 pathway in ADEV-induced protection [77].

miRNAs enriched in ADEVs can also interact with microglia. An in vitro study assessed the effect of EVs from morphine-treated astrocytes (morphine-ADEVs) on microglial phagocytosis unravelling a new mechanism of intercellular communication [78]. Morphine-ADEVs released a higher number of EVs compared to controls, through a mechanism induced by mu opioid receptor on astrocyte membranes. Morphine-ADEVs were shown to be taken up by microglia, and to reach their endosomes, where they could release their miRNA cargo, enriched in several miRNA that showed TLR7/8 motif. These miRNAs were able to bind the endosomal Toll-like receptor TLR7, thus activating NFkB, which rapidly upregulated the long intergenic noncoding RNA (lincRNA)-Cox2 [78]. LincRNA-Cox2 is a coactivator required for the transcription of late primary inflammatory-response genes and was described to downregulate the expression of microglial genes related to phagocytosis [79,80]. Intranasal delivery of EVs containing lincRNA-Cox2 siRNA was able to restore microglial phagocytic activity in mice treated with morphine. These findings have potential implications for the development of EV-loaded RNA-based drugs for the treatment of disorders in which microglial phagocytosis is impaired [78]. 

The generation of human neural cells from induced pluripotent stem cells (iPSC) allowed to demonstrate that specific genetic mutations may profoundly modify the generation of glial cell EVs, and their cargo. Astrocytes derived from iPSC of Amyotrophic Lateral Sclerosis (ALS) patients with the C9ORF72 mutation produced not only a smaller number of EVs, but a different miRNA content, which exhibited a detrimental effect on neuronal network and motor neuron survival in vitro [74]. The dysregulated miRNAs were predicted to target genes involved in axonal guidance and maintenance as well as adherens junctions. Among the downregulated miRNAs, the authors have highlighted the role of miR-494-3p, with its primary target being Semaphorin 3A [74], which was previously reported to be abundant in the ALS frontal cortex of human postmortem tissues [81]. Interestingly, miR-494-3p was also shown to be downregulated in the spinal cord tissues of ALS patients, although not accompanied by higher levels of Semaphorin 3, suggesting that the levels of Semaphorin 3 can also depend on the regulation by other cells and other miRNAs. In vitro, however, the treatment of mouse motor neurons with the miR-494-3p mimic reduced the levels of Semaphorin 3A. Furthermore, the treatment of motor neurons with the medium of C9 iPSC-derived astrocytes that were supplemented with a miR-494-3p mimic has rescued the neurite length and number of nodes per cell when compared with the non-supplemented control media, indicating that miR-494-3p level in astrocytes can directly influence the rescue and structure of neurons, and thus, their function [74]. A summary of the studies described in this paragraph is reported in Table 2.

### 4.3. Effect of Oligodendroglial EV-Derived ncRNAs on Neuroinflammation

In oligodendrocytes, EV secretion can be stimulated by exposure to neuronal signals, such as glutamate and other AMPA and NMDA agonists, in a Ca^2+^ entry dependent manner [82]. EVs from oligodendrocytes are mainly internalized by microglia, sustaining chaperone activity and autophagy and as well as by reducing oxidative stress-induced apoptotic cell death [83], and neurons, exerting protective effects against oxidative and nutrient deprivation stress [82].

**Table 2:** Summary of glial EV-derived ncRNAs, their targets, and effects.

Source	Recipient Cells	ncRNAs	ncRNA TARGETS	Mechanism of Action	Reference
Rat hippocampal and cortical microglia	Rat hippocampal neurons	miR-146a-5p miR-181a miR-223-3p	Nlg1, GluR2, and GluN2B	Synaptic strength impairment	[59]
BV-2 microglial cell line	Rat cortical oligodendrocytes	miR-23a-5p	Olig3	Survival and maturation under oxygen-glucose deprivation (OGD), and white matter repair after mouse tMCAO ischemic model	[60]
BV-2 microglial cell line	Mouse cortical neurons	miR-124-3p	PDE4B	Inhibited neuroinflammation in s scratch-injury model	[46]
BV-2 microglial cell line	Mouse cortical astrocytes	miR-124	STAT3	Reduction of glial scar under oxygen-glucose deprivation, and reduction of infarct volume after mouse tMCAO ischemic model	[61]
Microglia derived EVs from rat serum	Neurons from rat hippocampal slices	miR-146a-5p circANKS1B	KLF4 and CDKL5	Suppression of neurogenesis, and synaptic impairment in chronic unpredictable mild stress (CUMS) model	[62]
BV-2 microglial cell line	HT22 mouse hippocampal neuronal cell line, mouse neurons	miR-124-3p	Rela (p65)	Inhibition of neurodegeneration and improvement of cognitive function in repetitive mild traumatic brain injury (rmTBI)	[49]
BV-2 microglial cell line	Mouse cortical neurons	miR-137	Notch1	Increased viability and reduction of apoptosis after mouse transient middle cerebral artery occlusion (tMCAO) ischemic model	[65]
BV-2 microglial cell line	HT22 mouse hippocampal neuronal cell line, mouse neurons	miR-124-3p	FIP200	Inhibition of autophagy	[68]
Rat cortical astrocytes	Mouse hippocampal and cortical neurons	miR-125a-5p miR-16-5p	NTRK3	Reduction of dendritic growth and complexity in Parkinson’s disease (PD) model	[70]
Rat cortical astrocytes	Rat dopaminergic neurons	miR-34a	Bcl-2	Enhanced vulnerability of dopaminergic neurons to neurotoxin in PD model	[73]
Human induced astrocytes	Mouse Hb9-GFP motor neurons	miR-494-3p	Semaphorin 3A	Sustainment of survival and protection of neurites	[74]
Mouse cortical astrocytes	HT22 mouse hippocampal neuronal cell line	miR-190b	ATG7	Reduction of apoptosis and inhibition of autophagy after OGD	[75]
Human astrocytic cell line A172, mouse whole brain astrocytes	BV-2 microglial cell line, Mouse cortical microglia	lincRNA-Cox2	Toll-like receptors and NFκB	Restores phagocytic activity in vitro and in mice treated with morphine	[78]
Human cortical astrocytes	Human neurons	NKILA	miR-195, NFκB and NLRX1	Increases cell proliferation; attenuation of apoptosis and injury in mice submitted to TBI	[77]

A recent study showed that systemic administration of EVs from mature oligodendrocytes, which are enriched in myelin antigens, improved the clinical score and increased the survival rate in mice models of experimental autoimmune encephalitis, reducing T cell infiltration, demyelination, and axonal damage [84]. Despite the oligodendrocyte ncRNA landscapes being known [9,50,85], their presence in oligodendrocyte-derived EVs and their function in cell-to-cell communication are still unexplored fields. However, results from ectopic expression studies of oligodendrocyte-specific ncRNAs in other neural cells suggest that they may play a key role. For example, overexpression of miR-219-5p and miR-338-3p, which guide oligodendrocyte differentiation and promote myelin repair after a demyelinating insult [86], decreased the expression of pro-inflammatory mediators in microglia and reduced astrocyte activation [87]. Moreover, miR-219 exerted a neuroprotective effect on glutamate-induced neurotoxicity when overexpressed in hippocampal neurons, by inhibiting caspase-3 activity and regulating CaMKIIγ [88]. Copy number variants in miR-138, another oligodendrocyte-enriched miRNA, was found to be a potential risk factor for early-onset Alzheimer’s disease, regulating different biological pathways implicated in amyloid and tau metabolism [89]. Accordingly, overexpression of pre-miR-138 in neurons of wild type mice has shown a negative impact on learning, memory, and anxiety, production of Aβ42 in the hippocampus and brain integrity [90]. Altogether these studies suggest that investigating the role of oligodendroglial EV-derived ncRNAs in physiological, degenerative, and inflammatory conditions could help to better understand the pathogenic mechanisms at the basis of neurological disorders.

## 5. Conclusions

The studies discussed in this review highlighted that, when stored in EVs, ncRNAs can be transferred to recipient cells and modulate their functions. These mechanisms are not only involved in cellular homeostasis, but also in the pathogenesis of neurodegenerative conditions, where alteration in extracellular ncRNA levels could contribute to either propagate neuroinflammatory waves or promote reparative functions.

It is worth noting that most of the experiments designed to decipher the mechanisms of cell-to-cell communication are based on in vitro models, in which ncRNAs or EVs are used in controlled conditions, and they might not adequately reproduce the complex environment of the CNS. However, these proof-of-concept experiments are fundamental to assess the potential contribution of specific ncRNAs in the regulation of the pathophysiological processes based on cell-to-cell communication mechanisms. The next challenge is to translate the current knowledge into clinics, for example using RNA-targeting or RNA-based drugs to restore the physiological communication.

Importantly, brain-derived ncRNAs may also represent useful peripheral biomarkers of CNS diseases; indeed, the same EVs secreted by CNS cells to communicate to each other can also be found in body fluids. However, although the recent advances in analytical and omics techniques allows to selectively identify neuron- and glia-derived EVs in plasma and cerebrospinal fluid and to characterize their cargo [91,92,93,94], the correlation between tissue ncRNAs and peripheral ncRNAs stored in EVs is still elusive, and the clinical significance in human disease is not fully clarified.

## Data Availability

Not applicable.
